# Genetic Structure of Wild Germplasm of Macadamia: Species Assignment, Diversity and Phylogeographic Relationships

**DOI:** 10.3390/plants9060714

**Published:** 2020-06-03

**Authors:** Thuy Mai, Mobashwer Alam, Craig Hardner, Robert Henry, Bruce Topp

**Affiliations:** Queensland Alliance for Agriculture and Food Innovation, The University of Queensland, Brisbane, QLD 4072, Australia; m.alam@uq.edu.au (M.A.); c.hardner@uq.edu.au (C.H.); robert.henry@uq.edu.au (R.H.); b.topp@uq.edu.au (B.T.)

**Keywords:** genetic diversity, DArT markers, macadamia, dendrogram, principal coordinate analysis, population structure, population genetics, wild species

## Abstract

Macadamia is an Australian native rainforest tree that has been domesticated and traded internationally for its premium nuts. Common cultivars rely upon a limited gene pool that has exploited only two of the four species. Introducing a more diverse germplasm will broaden the genetic base for future crop improvement and better adaptation for changing environments. This study investigated the genetic structure of 302 accessions of wild germplasm using 2872 SNP and 8415 silicoDArT markers. Structure analysis and principal coordinate analysis (PCoA) assigned the 302 accessions into four distinct groups: (i) *Macadamia integrifolia*, (ii) *M. tetraphylla*, and (iii) *M. jansenii* and *M. ternifolia*, and (iv) admixtures or hybrids. Assignment of the four species matched well with previous characterisations, except for one *M. integrifolia* and four *M. tetraphylla* accessions. Using SNP markers, 94 previously unidentified accessions were assigned into the four distinct groups. Finally, 287 accessions were identified as pure examples of one of the four species and 15 as hybrids of *M. integrifolia* and *M. tetraphylla*. The admixed accessions showed the highest genetic diversity followed by *M. integrifolia*, while *M. ternifolia* and *M. jansenii* accessions were the least diverse. Mantel test analysis showed a significant correlation between genetic and geographic distance for *M. integrifolia* (r = 0.51, *p* = 0.05) and a positive but not significant correlation for *M. tetraphylla* (r = 0.45, *p* = 0.06). This study provides a population genetics overview of macadamia germplasm as a background for a conservation strategy and provides directions for future macadamia breeding.

## 1. Introduction

The genus *Macadamia* belongs to the Proteaceae family and is composed of four species: *Macadamia integrifolia* Maiden and Betche, *M. jansenii* C.L. Gross and P.H. Weston, *M. ternifolia* F. Muell and *M. tetraphylla* L.A.S. Johnson. The natural distribution of the four species is in the subtropical rainforest from south-east Queensland (QLD) to north-east New South Wales (NSW), Australia [1,2]. *M. integrifolia* and *M. ternifolia* occur in south-east QLD, while *M. tetraphylla* is distributed mainly in northern NSW, with some extension into south-east QLD [3]. Overlapping zones exist between *M. integrifolia* and *M. tetraphylla* and between *M. integrifolia* and *M. ternifolia*, with natural hybridisation occurring in these zones [4]. The fourth species, *M. jansenii*, has been found only in a single location in Bulburin (QLD) that is 180 km north from the nearest population of *M. integrifolia* [5]. Of these four species, *M. integrifolia* and *M. tetraphylla* produce edible nuts, and hence, most of the commercial cultivars belong to either of these two species or their hybrids [6]. The other two species, *M. jansenii* and *M. ternifolia*, have not been used in directed breeding, due to their bitter inedible nuts containing high levels of cyanogenic glycoside [7,8,9]. Most current commercial varieties appear to be two to four generations from the wild [1]. The majority of global macadamia production relies upon the cultivars from the Hawaiian breeding program, which is comprised mostly of a limited gene pool of *M. integrifolia* [10,11]. However, wild genetic resources have the potential to provide parents with desirable traits, including small tree size, nuts with thinner shells, resistance/ tolerance to biotic and abiotic factors, etc. [12] Exploring the genetic potential of the wild germplasm will facilitate further exploitation of these resources in trait improvement.

The study of genetic diversity and population structure can determine the potential of wild germplasm in future breeding. The genetic structure is formed over time due to the multiple actions of migration, selection, mutation, and genetic drift [13], as well as the mode and method of reproduction. A diverse species has the opportunity for selection of the fittest alleles while low diversity leads to the risk of extinction [14]. Broadening the genetic base of breeding material requires the identification of diverse parents for crossing with the cultivated crop [14]. Understanding the genetic relationship among the parents is essential to avoid inbreeding depression, particularly for the improvement of complex traits. Therefore, knowledge of the genetic divergence is a prerequisite to maximise heterosis in the breeding progeny [15]. The history of world macadamia breeding is very short, and the existing cultivars are only a few generations from the wild germplasm growing naturally in the rain forest. Recently, a chloroplast genome sequencing project on wild and cultivated germplasm indicated that all major Hawaiian cultivars share a single chlorotype probably derived from a small sample form single location [16]. As current macadamia plantations are mostly dependent on Hawaiian cultivars, this limited sampling suggests there is an opportunity for future genetic improvement by exploiting the diverse genetic resource. In addition, the genetic base can be expanded in breeding material by selecting diverse parents from wild germplasm for crossing with the cultivated crop.

Molecular markers are considered as the most suitable tool to estimate genetic diversity, due to their polymorphic nature and independence to environmental effects [17]. Several molecular marker systems have been developed to study in macadamia and only a few of them were used for the genetic characterisation of wild germplasm. An isozyme-based study was conducted by Aradhya et al. [18] to identify the genetic relationships among 40 cultivars (35 *M. integrifolia*, three *M. tetraphylla*, and two hybrids) and three *M. ternifolia* accessions. Mast et al. [19] studied the relationships among the four *Macadamia* species and their wild relatives, using three cpDNAs (*matK, atpB*, and *ndhF*), three nDNA (*waxy* loci *1* and *2*, and *PHYA*) genomic regions. However, this study used only one accession per species. The genetic structure of a large number of wild germplasm accessions was studied by Peace [4] using low throughput RAF (randomly amplified DNA fingerprinting, dominant) and RAMiFi (randomly amplified microsatellite fingerprinting, co-dominant) markers. All these marker systems have limitations, including a low total number of markers, low marker density, and low genome coverage, and hence, are seldom used in genomic studies. SSRs (simple sequence repeat markers or microsatellite) are considered as one of the best marker systems for genetic studies, with many advantages, such as stability, PCR-based amplification, and relatively low cost [17]. SSRs were used in the genetic diversity study of wild *M. integrifolia* [20] and wild *M. tetraphylla* populations [21,22]. However, there is a limited number of SSR primers available for macadamia, particularly those that successfully amplify across species [23], and as such, may not be effective for a large-scale genetic study of all four wild species.

The rapid advancement of next generation sequencing technology (NGS) enables the discovery of high-throughput and cost-effective molecular marker systems. Using NGS technology, Diversity Array Technology (DArT) developed a marker system that facilities affordable whole-genome level genetic characterisation. DArT has been successfully applied for the genetic diversity, population structure and genetic mapping studies of many crop species [24,25,26]. Recently, Alam et al. [27] used 11,526 silicoDArTs and 3956 SNPs to study the genetic diversity and population structure of 80 macadamia cultivars. O’Connor et al. [28] reported the genetic diversity, population structure and linkage disequilibrium of 295 seedling progenies from 29 selected parents, using 16,171 silicoDArTs and 4113 SNPs. These studies suggest that DArTseq markers could be applied for genomic studies in the wild germplasm of macadamia.

In this study, for the first time, we used high-throughput DArTseq platforms for the genetic characterisation of a large number of wild accessions of macadamia. The aims were to: (1) assess the population structure of wild macadamia germplasm, (2) explore the genetic diversity among the accessions within species, and (3) determine the relationship between genetic and geographic distance within *M. integrifolia* and *M. tetraphylla*.

## 2. Results

### 2.1. Quality of DArTseq Markers

**Figure 1:**
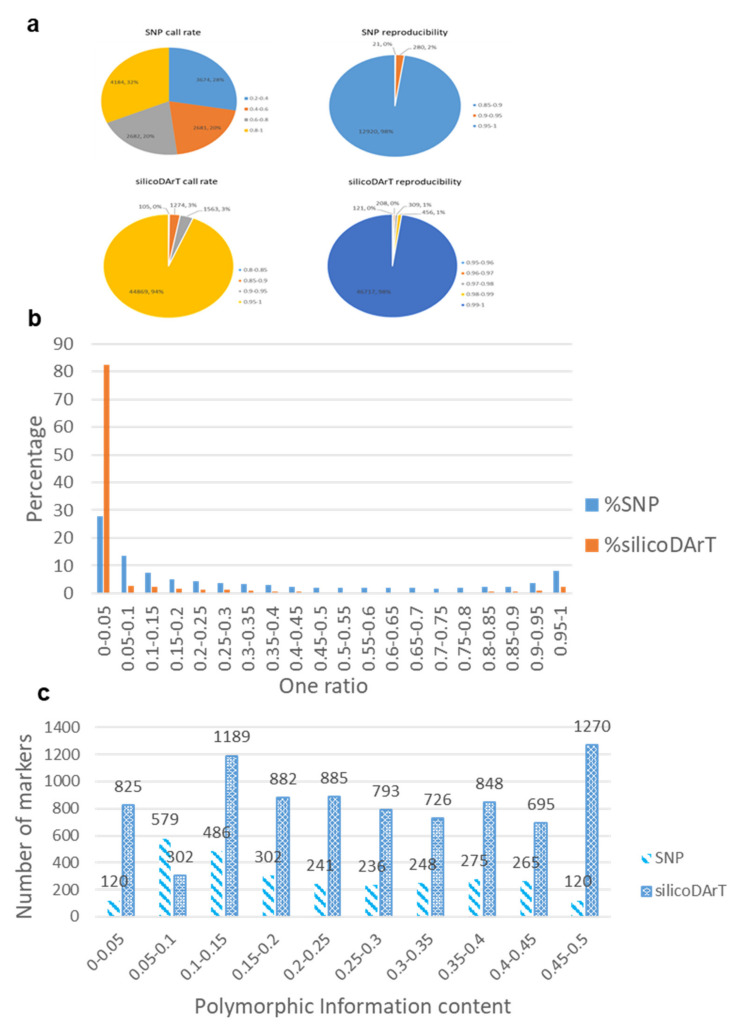
Distribution of SNP and silicoDArT marker data for several quality parameters. (**a**) call rate and reproducibility; (**b**) one ratio and (**c**) polymorphic information content (PIC) value.

DArTseq platforms generated 13,221 SNP and 47,811 silicoDArT markers. The call rates of SNP markers varied from 0.20 to 1.00, with an average of 0.62 (Supplementary Materials Table S1). Of the 13,221 SNPs, the call rate of 4184 markers (32%) was > 0.80 (Figure 1a). The reproducibility of SNPs varied from 0.86 to 1.00, and most of them (98%) were over 0.95 (Figure 1a). The call rate of silicoDArT markers varied from 0.81 to 1.00 (Table S1). The average call rate was very high (>0.99), with 94% of markers having a call rate over 0.95 (Figure 1a). The range of reproducibility was 0.95 to 1.00, in which 98% of the markers had very high value (0.99) of reproducibility. Mean one ratio was higher in SNPs (0.32) than in silicoDArT markers (0.08) (Table S1). Most silicoDArTs (82%) had one ratio below 0.05, compared with only 28% of SNPs (Figure 1b). Considering the quality parameters: call rate (>0.80), reproducibility (>0.95) and one ratio (>0.05), 2872 SNPs and 8415 silicoDArTs were retained for further analysis (Tables S2 and S3). The remaining markers had PIC values from 0 to 0.5 for both SNPs and silicoDArTs (Figure 1c). Mean PIC was 0.26 for silicoDArTs and 0.22 for SNPs. Only 120 of the SNPs (4%) had low PIC (<0.05), compared with 825 (9.8%) of silicoDArTs (Figure 1c).

### 2.2. Population Assignment

The K and Q values from STRUCTURE analysis were used for the assignment of individual accessions in each species/hybrid group. Population clusters in PCoA validated the species representation.

**Figure 2:**
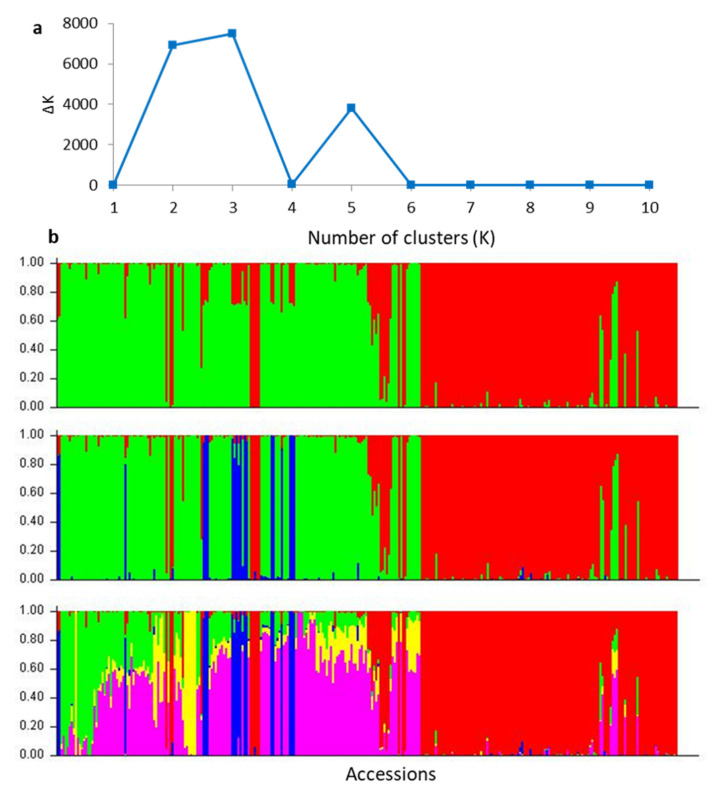
Population structure of 302 accessions based on 2872 SNPs, as inferred by STRUCTURE. (**a**) Best value of K based on Evanno’s ΔK; (**b**) Individual membership proportions (Q value) in two, three and five clusters, with each cluster represented by a colour block. Each vertical line represents one accession. The accessions were sorted on the x-axis by latitude from north to south.

The ΔK from the STRUCTURE analysis of SNP markers was significant when K = 2, 3 and 5, with a peak at K = 3 (Figure 2a). The optimal peak at K = 3 suggested that the 302 accessions in the germplasm were derived from three distinct clusters, as represented by different colours in the structure analysis (Figure 2b, K = 3), here named Cluster I (blue), Cluster II (green) and Cluster III (red). Cluster I was composed of 18 accessions, including eight previously labelled as *M. ternifolia*, two as *M. jansenii*, and eight as undefined species (Table S4). These undefined eight accessions, originally labelled as mixed/hybrid populations, were collected from the natural distribution of *M. ternifolia*. Considering this distribution and their morphological appearance (Thuy Mai, pers. observations), these accessions were classified as *M. ternifolia*. Cluster II contained 99 predefined *M. integrifolia* and 36 accessions of previously undefined species. Cluster III was comprised of 94 predefined *M. tetraphylla* and 38 undefined species. There were 17 accessions, including one predefined *M. integrifolia*, four predefined *M. tetraphylla*, and 12 accessions from planted/unknown/mixed populations that showed the genetic admixture (hybrid) of clusters. For example, the accession “M034”, which was previously labelled as *M. integrifolia*, consisted of 6% of Cluster II (predominantly *M. integrifolia*), 16% of Cluster III (predominantly *M. tetraphylla*), but 78% of Cluster I (predominantly *M. jansenii* and *M. ternifolia*). Two previously labelled *M. tetraphylla* accessions (“M265” and “M266”) were identified as admixtures of Cluster II & III. The accession “M265” was composed of 61% of Cluster II and 39% of Cluster III, and accession “M266” was composed of 52% of Cluster III.

The pattern displayed for K = 2 (Figure 2b) grouped the accessions of three species, *M. jansenii*, *M. ternifolia* and *M. tetraphylla*, into one cluster, and separated *M. integrifolia* accessions in another cluster. The pattern displayed for K = 5 (Figure 2b) still did not separate the accessions of the two species *M. jansenii* and *M. ternifolia* but divided the accessions of *M. integrifolia* into smaller sub-clusters.

**Figure 3:**
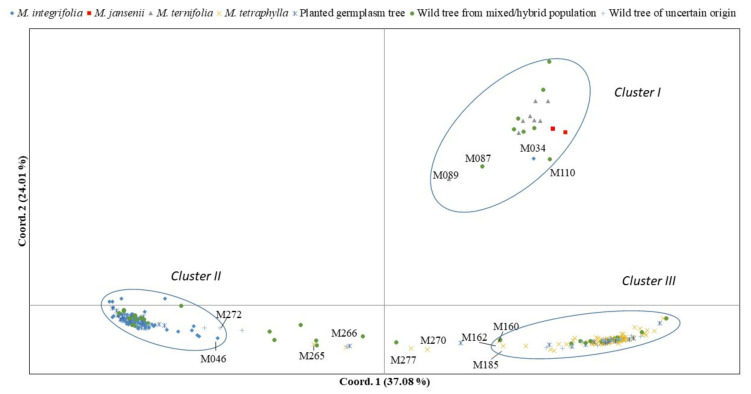
Principal coordinate analysis (PCoA) of the 302 accessions based on 2872 SNP markers, showing the three distinct groups and the admixtures. The first two coordinates of PCoA explained 34.06% of the total variation.

Principal coordinate analyses (PCoA) of SNP markers via distance matrix with data standardization identified three distinct groups of the four species (Figure 3). This result was consistent with the result of STRUCTURE analysis at K = 3. The first two coordinates of PCoA explained 61.09% of total variation in SNPs. The accessions of two species *M. ternifolia* and *M. jansenii* formed Cluster I. Cluster II was formed by the accessions of *M. integrifolia* and Cluster III included the accessions of *M. tetraphylla*. The accession “M034”, which was assigned as admixture in STRUCTURE analysis, was clustered in the *M. jansenii*/*M. ternifolia* group and shows a close relationship with two *M. jansenii* accessions. The accession “M160”, which was also assigned as admixture in STRUCTURE analysis composing 78% of Cluster III, was clustered in the *M. tetraphylla* group.

Finally, based on the STRUCTURE analysis and PCoA, we assigned 302 wild accessions into 287 pure accessions, representing the four distinct species, and 15 admixtures. Pure accessions are composed of 135 *M. integrifolia*, 133 *M. tetraphylla*, and 19 *M. ternifolia*/*M. jansenii* (Table S4).

### 2.3. Genetic Diversity

We estimated the genetic diversity parameters among 302 accessions, using both SNP and silicoDArT markers (Table 1). For SNP markers, the number of effective alleles (*Ne*) ranged from 1.08 to 1.34, with the lowest in *M. ternifolia*/*M. jansenii* group and the highest in admixture. Shannon’s index (*I*) ranged from 0.11 (*M. ternifolia*/*M. jansenii*) to 0.33 (admixture), with a mean of 0.23. In all clusters, the observed heterozygosity (*Ho*) was smaller than the expected heterozygosity (*He*). *He* was highest in admixture (0.21) and lowest in *M. ternifolia*/ *M. jansenii* (0.07), with a mean of 0.15. Interestingly, accessions from the *M. integrifolia* group showed the highest percentage (86.53%) of polymorphism (%P), followed by admixture (74.93%), *M. tetraphylla* (71.5%), *M. ternifolia*/*M. jansenii* (32%) groups. Similar results were also observed for silicoDArT markers (Table 1).

Nei’s genetic distance (D), based on SNP markers (Table 2), ranged from 0.06 between admixtures and *M. integrifolia* to 0.27 between *M. integrifolia* and *M. ternifolia*/*M. jansenii* groups. *M. tetraphylla* accessions shows lower genetic distance with *M. integrifolia* (D = 0.2) than that of *M. ternifolia*/*M. jansenii* accessions (D = 0.23). Hence, the admixture accessions showed almost similar genetic distance with *M. integrifolia* (0.06) and *M. tetraphylla* (0.07) germplasm. Although the estimated value of genetic distance using silicoDArT markers was lower than that of SNPs, the genetic relationship between species was similar in both marker systems (Table 2).

The analysis of molecular variance (AMOVA) showed a higher proportion of variance detected within species than among clusters (Table 3). For SNPs, the percentage of genetic variation within species (55%) was higher than that among species (45%). A similar pattern of genetic variation was observed using silicoDArT markers (Table 3).

### 2.4. Phylogeographic Relationships among the Accessions of M. integrifolia and M. tetraphylla

**Figure 4:**
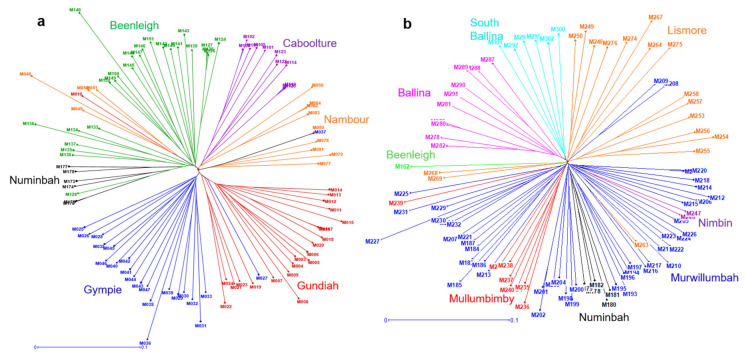
Unweighted neighbour-joining dendrograms using 2872 SNP markers, showing the genetic relationships among (**a**) 99 *M. integrifolia* accessions and (**b**) 94 *M. tetraphylla* accessions. Localities are represented by different colours.

We identified the genetic relationships within each species of *M. integrifolia* and *M. tetraphylla* presented in the dendrogram (Figure 4). Most accessions from the same locality grouped together, although some accessions from a locality clustered with accessions from other localities. For example, within *M. integrifolia*, the accession “M010” from Gundiah and four accessions from Nambour grouped with Beenleigh accessions (Figure 4a). Two accessions, “M027” and “M037”, from Gympie grouped with accessions from Gundiah and Nambour, respectively. Within *M. tetraphylla*, the accession “M263” from Lismore grouped with Murwillumbah, while the accessions “M208” and “M209” from Murwillumbah grouped with Lismore (Figure 4b). Some of the accessions showed an unexpectedly variable branch length compared to other accessions from the same cluster. Accessions with the longest branches represent the most diverged accessions within the population. For example, accession “M148” had ~50% longer branch than other Beenleigh accessions. Similarly, accessions “M036”, “M048”, “M136” and “M008” of *M. integrifolia*, and “M227”, and “M267” of *M. tetraphylla* had significantly longer branches.

To explore the genetic basis of the geographic relationship we calculated the correlation between the genetic distance and geographic distances of the localities for each species. The pairwise genetic distance among the accessions of six localities of *M. integrifolia* (Table S5) varied from 0.019 to 0.055, with an average of 0.034. The genetic distance between Gundiah and Gympie was the closest (0.019), while it was the farthest (0.055) between the accessions of Numinbah and Caboolture. The pairwise genetic distances among the accessions of *M. tetraphylla* localities (Table S6) were higher than that of *M. integrifolia*. The range of variation in *M. tetraphylla* was 0.013 to 0.151, with a mean of 0.063. Accessions from locality Murwillumbah and Lismore showed the closest genetic distance (0.013), while the highest (0.151) was observed between the accessions of Beenleigh and Nimbin.

**Figure 5:**
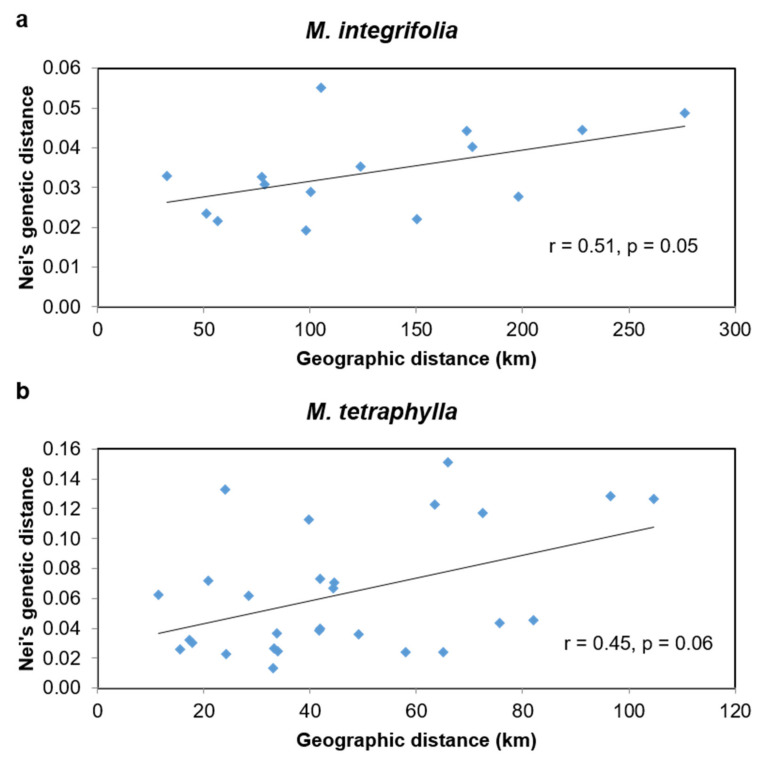
Correlation between genetic and geographical distance among (**a**) six localities of *M. integrifolia*, and (**b**) eight localities of *M. tetraphylla*, based on a Mantel test at 999 random permutations.

The Mantel test analysis showed a significant correlation between genetic distance and geographic distance (r = 0.51, *p* = 0.05) among *M. integrifolia* localities (Figure 5a). Meanwhile, the correlation between genetic and geographic distance among *M. tetraphylla* localities was positive but not significant (r = 0.45, *p* = 0.06) (Figure 5b).

## 3. Discussion

### 3.1. Species Assignment of Wild Macadamia Germplasm

Population structure and PCoA of DArTseq based SNP markers facilitated the assignment of individuals in corresponding species or hybrid groups. Our species classification of each accession matches well with the field note and previous DNA study [4]. Results from this study confirmed that most of the previous phenotypic characterisations were successful for species’ identification of wild germplasm.

All but one *M. integrifolia* accessions were clustered together in the same group. “M034”, an accession from the “Mooloo” region of the “Gympie” locality, was identified as *M. ternifolia*/*M jansenii*, although it was previously recorded as *M. integrifolia*. A co-investigation of wild germplasm using 15 SSR markers identified that the same accession “M034” is a clone of another accession (X-CANB896104) from Canberra Botanic Garden. Interestingly, accession “X-CANB896104” is recorded as a cutting from wild *M. ternifolia* from Mary Cairncross Park, Maleny (Cathy Nock., pers communication). Further phenotypic characterisation confirmed that “M034” is a *M. ternifolia*. Among the 99 accessions of *M. tetraphylla*, four accessions were assigned as hybrid of *M. tetraphylla* and *M. integrifolia*. Accessions “M265” and “M266” shared almost 50% from each species, whereas “M270” and “M277” had ~30% from *M. integrifolia* genotype and ~70% *M. tetraphylla* (Figure 3, Table S3).

This study clearly demonstrated that the accessions of *M. tetraphylla* and *M. integrifolia* formed two distinct populations. The accessions of *M. jansenii* and *M. ternifolia* clustered together, although they were collected from geographically distant locations. The close relationship between *M. jansenii* and *M. ternifolia* is supported by the previous molecular studies [29,30]. These findings indicate that the accessions of both *M. jansenii* and *M. ternifolia* may have the same genetic lineage, or that one may be an ancestor of the other. Possibly, these two small groups of populations may have separated due to past climatic extremes and adapted as small groups in two distinct locations. It is to be noted that there may be some sampling effect of the small number of accessions of *M. jansenii* (*n* = 2) and *M. ternifolia* (*n* = 16) in our study. Though *M. jansenii* and *M. ternifolia* differ in a few simple traits including leaf tip, leaf serration and flower colour, they are morphologically similar, both with small tree size, and small and bitter nuts [9,31]. However, there may be further debate on the species differentiation of these two small populations. Investigation on evolutionary genetics and time divergence on four *Macadamia* species can be used to confirm the speciation of wild macadamia germplasm.

The species status of previously unidentified accessions, including those of unknown origins, planted germplasm and mixed/hybrid populations were resolved. Out of 94 unidentified accessions, 36 were assigned as *M. integrifolia*, 39 as *M. tetraphylla*, 8 as *M. ternifolia* and 11 as hybrids/admixtures of *M. integrifolia* and *M. tetraphylla* (Table S5). The genotypic classification was consistent with our field observation on phenotypic characteristics of representative species (Thuy Mai, pers communication) The species composition of many modern and heritage cultivars is uncertain [1], but this finding supports the potential of SNP markers to resolve their species status.

Some accessions we identified as *M. tetraphylla* had been collected from further north than the accepted distribution of this species. These were the accessions “M056”, and “M057” from population 16 (Palmwoods, Nambour QLD), accession “M054” from population 106 (Mapleton Kenilworth, Nambour QLD) and five accessions from population 36 (Mount Glorious, Caboolture QLD). However, these populations were noted at the time of collection as planted or uncertain populations, and it seems highly likely that their locations were the result of human activity.

### 3.2. Genetic Diversity of the Four Macadamia Species

In this study, we developed new knowledge on genetic diversity in wild accessions by using high-throughput silicoDArT and SNPs marker. In both types of markers, the average expected heterozygosity (*He*) across the wild germplasm and clusters forming “pure” species was lower than in previous genetic studies of macadamia cultivars [21,22,23,32]. However, the cluster of hybrid accessions from wild *M. integrifolia* and *M. tetraphylla*, which is composed of a small number of accessions (*n* = 15), showed greater gene diversity than previous studies on cultivars and pure wild species in the current study. This result indicates that crossing between two species can be conducted, to increase genetic diversity in the future breeding program.

Our results suggested that *M. integrifolia* and *M. tetraphylla* contained two- to three-fold greater diversity than the *M. jansenii* and *M. ternifolia* cluster. Population size has a significant effect on genetic diversity [33]. Generally, smaller population size leads to lower genetic diversity [34]. Extinction and contraction of species’ distribution during successive ice ages has resulted in reduced population size and resultant diversity bottlenecks in other Australian flora, such as *Acacia, Banksia, Eucalyptus* etc. [35]. Certainly, *M. jansenii* formed a very small population, with less than 100 individuals comprising the whole species [5,9]. In this study, only a small number of *M. jansenii* (*n* = 2) and *M. ternifolia* (*n* = 17) have been tested. The lower diversity of *M. jansenii* and *M. ternifolia* in our study may be the result of small populations. An investigation with larger sample numbers from diverse distributions of *M. jansenii* and *M. ternifolia* should be conducted, to define their genetic diversity more completely and accurately.

### 3.3. Phylogeographic Relationship among the Accessions of M. integrifolia and M. tetraphylla

**Figure 6:**
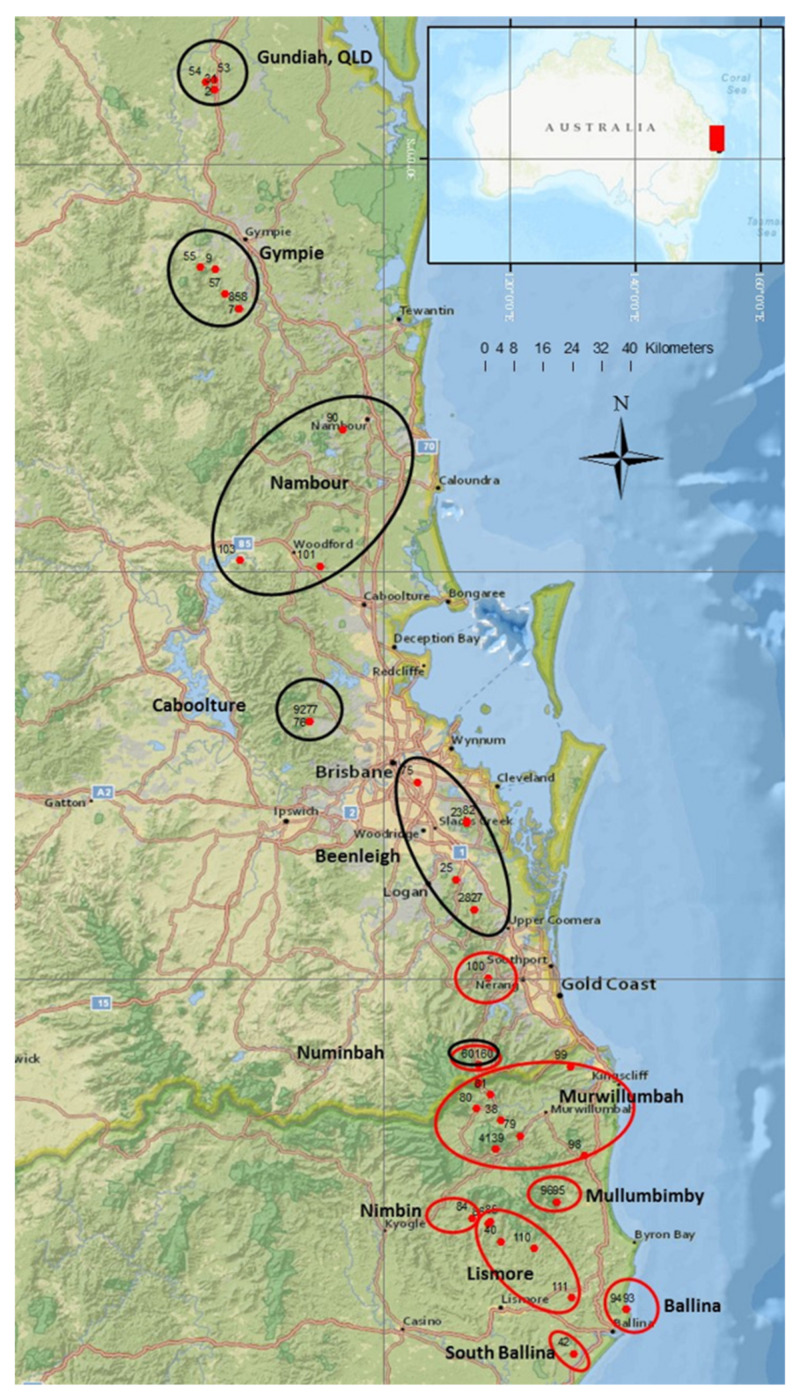
Map showing origins of representative wild-germplasm accessions of *M. integrifolia* and *M. tetraphylla*. The black circles represent six localities of *M. integrifolia* population, and the red circles represent eight localities of *M. tetraphylla* populations.

Geographical distance is one of the major contributing factors in species differentiation. Knowledge of the genetic structure of a species over its geographic distribution is important to develop an understanding of the evolutionary processes [36]. In this study, the neighbour-joining tree, based on dissimilarity matrix (Figure 6), showed that the accessions from the same geographical area appeared to be grouped together. However, some accessions (e.g., “M010”, “M027”, “M037”, “M263”, “M208”, “M209”) were found to be clustered with accessions of different geographical regions (Figure 6). This result was supported by a previous study on chloroplast genome sequence, where Nock et al. [16] reported the relocation of some accessions within the *M. integrifolia* germplasm. Since the gene flow for both species is restricted within a short distance (~50 km) [1], the impact of environmental parameters, such as water, gravity, and animals like rodents [37], or a result of human activity on the seed transportation [16], could be considered as the cause of relocation.

Phylogeographic analysis of *M. integrifolia* accessions revealed a significant positive correlation between genetic and geographic distance (r = 0.51, *p* = 0.05). This correlation was higher than a previous study using RAF markers [4] (r = 0.16, *p* = 0.016), and in almond germplasm (r = 0.173, *p* = 0.226) [38]. Based on chloroplast genome study, Nock et al. [16] also found a latitudinal population structure among these accessions. We also observed a positive relationship between genetic and geographic distance among the accessions of wild *M. tetraphylla*. Although, this correlation (r = 0.45) is non-significant, it is higher than that found by Peace [4] (r = 0.13). The non-significant phylogeographic relationship in *M. tetraphylla* suggested that geographic distance may not be the main factor influencing the genetic distance between populations of this species, although geographical boundaries, low gene flow and genetic drift are typically key factors explaining genetic differentiation in fragmented populations [39].

The phylogenetic trees (Figure 4) of *M. integrifolia* and *M. tetraphylla* provided an indication of ancestral lineage of the accessions of each species. Interestingly, the root of most of the accessions of both species originated from the population of Numinbah (Figure 4). It is to be noted that Numinbah is an overlapping region of both species, and its surrounding regions are the sources of mixed/hybrid population. We hypothesise that there is a possibility of early divergence of these two species at Numinbah. Later, smooth leaved *M. integrifolia* may have adapted to the north and serrated leaved *M. tetraphylla* may have adapted to the southern regions. Further genomic investigation with more accessions from Numinbah can explain the origin of these two species.

## 4. Materials and Methods

### 4.1. Collection of Plant Samples

The germplasm field trials were established in 2000 and 2001 in Tiaro, Queensland (QLD) and Alstonville, New South Wales (NSW), Australia. Wild accessions were collected from multiple geographical regions (Figure 6), covering the natural distribution of the four species. Ramets of each accession were propagated clonally as rooted cuttings. During the collecting trip, the germplasm collector (S. Faulkner) classified the populations as “wild”, “planted” (trees were cultivated from the local wild trees), “hybrid/mixed”, or “uncertain”. The accession(s) from each population may belong to more than one of these classifications; therefore, the original description for each accession was noted. Accessions of the rare species *M. jansenii* were added to the field trial in July 2011. From these collections, we studied 302 accessions, including 100 *M. integrifolia*, two *M. jansenii*, eight *M. ternifolia*, 98 *M. tetraphylla*, and 94 accessions of undefined species (from mixed/hybrid populations, uncertain origins or planted germplasm). These accessions originated from 75 populations (one to seven accessions per population, averaging 4.1) across 52 regions (one to three populations per region) from 14 localities (one to nine regions per locality) (Table S7). The accessions were ordered by latitude from north to south and coded from “M001” to “M302”.

### 4.2. DNA Extraction and Genotyping

Newly flushed young leaves were collected from the ex situ trials at Tiaro and Alstonville in December 2017 and placed in zipped plastic bags inside a cool box with ice blocks. The materials were stored in a cold room at 4 °C before transfer to DArT Pty Ltd. (Canberra, Australian Capital Territory, Australia), to perform DNA extraction. A total of 2–3 mg of leaf tissue from each sample was sub-sampled into a 1.1 mL microtube containing a disposable steel ball bearing. Leaf samples were crushed using a QIAGEN Tissue Lyser (Qiagen, Germany). A Freedom EVO robotic Tecan 100 (Tecan Group, Switzerland) was used for DNA extraction following an existing protocol recommended by DArT (https://www.diversityarrays.com/). DNA samples were incubated with loading dye at 37 °C for two hours and then checked for quality control on 0.8% agarose electrophoresis gel for 30 min at 100 V.

Accessions were genotyped for dominant silicoDArT and co-dominant SNP markers following an established protocol developed by Kilian et al. [40] An appropriate method (*Pst*I + *Hha*I) of complexity reduction was selected to detect DArTseq-based markers using next generation sequencing (NGS) technology. The silicoDArT and SNP markers were scored as a binary data format in which the score was “1” for presence, “0” for absence and “-“, for failure to score of a marker in the genomic representation of each sample. The full details of methodology of developing DArTseq-based markers for macadamia were previously described by Alam et al. [27] and O’Connor et al. [28]

### 4.3. Analysis of Processed Marker Data

DArTseq platforms generated SNP and silicoDArT markers. DArTsoft v7.4 software was used to automatically identify and score the polymorphic markers, using a proprietary marker calling algorithms. The quality of the markers was tested for call rate (%), reproducibility (%), one ratio, and polymorphic information content (PIC). The call rate determines the success of reading the marker sequence across the samples, and was estimated from the percentage of samples for which the score was either “0” (absence of marker) or “1” (presence of marker). The scoring of reproducibility involves the proportion of 138 technical replicates for which the marker score exhibited consistency. One ratio was determined as the proportion of samples for which genotypes were scored as “1”. PIC is the degree of diversity of the marker in the population and shows the usefulness of the marker for linkage analysis. Quality control and filtering were applied to both SNP and silicoDArT markers, including call rate (>80%), reproducibility (>95%), and one ratio (>0.05).

### 4.4. Analysis of Population Structure and Genetic Diversity

The population structure of the 302 accessions of four species was identified based on SNP markers by using the Bayesian model-based program STRUCTURE v2.3.4 [41,42,43,44]. A burn-in length of 10,000 cycles and Markov chain Monte Carlo (MCMC) of 50,000 runs were set for the structure analysis. Cluster values (K) ranging from one to 10 were performed, each with ten different iterations. Results from STRUCTURE were uploaded to Structure Harvester [45], through http://taylor0.biology.ucla.edu/structureHarvester/, to determine the optimum number of populations (best value of K) using ∆K value, as described by Evanno et al. [46] The individual ancestry proportion (Q value) at the best K value was determined for each accession. Based on Q values, the accessions were identified as pure or admixed species: accessions with Q value more than 0.8 were considered as “pure” and accessions with Q value less than 0.8 were assigned as “admixture” [14,47].

The genetic diversity and principal coordinate analysis (PCoA) were performed using GenAlEx v6.5.2 software [48]. Both SNP and silicoDArT markers were used to calculate the diversity parameters, including the mean number of alleles per locus (Na), number of effective alleles (Ne), observed and expected heterozygosity (Ho and He), Shannon’s information index of diversity (I) and the percentage of polymorphic loci (%P). Pairwise population matrix of Nei’s genetic distance [49] and the analysis of molecular variance (AMOVA) were also estimated.

### 4.5. Phylogeographic Relationships Analysis

*M. integrifolia* and *M. tetraphylla* are the two major species used in current breeding programs. In this study, we have identified a large number of accessions of *M. integrifolia* and *M. tetraphylla*. Therefore, a detailed study was undertaken on the genetic relationships and geographical diversity of those species. Only accessions identified as “pure” representatives of each species that were confirmed by structure analysis, including 99 *M. integrifolia* accessions from six localities (Figure 6, black circles), and the 94 *M. tetraphylla* accessions from eight localities (Figure 6, red circles), were used. The “pure” accessions collected from planted wild germplasm or unknown origin were not included. DARwin v6.0 software [50] was used to estimate pairwise Jaccard’s genetic dissimilarity indices using 2872 SNP markers. A dendrogram was constructed by clustering accessions, based on a dissimilarity matrix using the unweighted neighbour-joining method. Clade strength in the dendrogram was tested using 100 bootstraps.

The correlation between genetic distance and geographic distance was estimated. The Nei’s genetic distance matrix and the pairwise geographic distance among the localities were calculated using GenAlEx v6.5.2 [48]. The Mantel test was used to determine the correlation between genetic and geographic distance, using 999 random permutations.

## 5. Conclusions

This is the first study to investigate the genetic structure of a large collection of wild macadamia germplasm using thousands of high-throughput molecular markers. A total of 302 wild accessions were characterised, using 2872 SNP and 8415 silicoDArT markers. Our population structure and principal co-ordinate analyses identified three distinct populations, in which *M. jansenii* and *M. ternifolia* formed a single cluster. The Nei’s genetic distance analysis clearly demonstrated that *M. jansenii* and *M. ternifolia* are related and showed greater heterozygosity in *M. ternifolia* than in *M. jansenii*. However, the limited number of accessions available in this study from *M. jansenii* and *M. ternifolia* limits the strength of our conclusion on the diversity and population structure of these species. We suggest that further analysis with more accessions from these two species should be conducted, to increase understanding of genetic diversity and clarify their classification as distinct species. We observed the significant correlation between genetic and geographic distance among *M. integrifolia* populations. Additionally, we were able to confirm the species identity of unknown wild accessions and suggested the use of these markers to resolve the unclear species composition of domesticated cultivars.

## Figures and Tables

**Table 1:** Genetic diversity parameters for Macadamia accessions based on SNP and silicoDArT markers. N = number of accessions, Na = number of different alleles, Ne = number of effective alleles, I = Shannon’s information index, Ho= observed heterozygosity, He = expected heterozygosity, %P = percentage of polymorphic loci, SE = standard error.

Group		N	Na	Ne	Ho	He	I	%P
**SNPs**								
*M. ternifolia*/*M. jansenii* (Cluster I)	Mean	19	1.28	1.08	0.04	0.07	0.11	32.00
	SE		0.01	0.01	0.00	0.00	0.00	
*M. integrifolia* (Cluster II)	Mean	135	1.87	1.28	0.12	0.18	0.29	86.53
	SE		0.01	0.01	0.00	0.00	0.00	
*M. tetraphylla* (Cluster III)	Mean	133	1.72	1.20	0.08	0.12	0.20	72.32
	SE		0.01	0.01	0.00	0.00	0.00	
Admixture	Mean	15	1.75	1.34	0.18	0.21	0.33	74.93
	SE		0.01	0.01	0.00	0.00	0.00	
Total	Mean	302	1.66	1.22	0.11	0.15	0.23	47.20
	SE		0.01	0.00	0.00	0.00	0.00	19.30
**silicoDArTs**								
*M. ternifolia*/*M. jansenii* (Cluster I)	Mean	19	0.58	1.11	-	0.07	0.11	24.01
	SE		0.01	0.00	-	0.00	0.00	
*M. integrifolia* (Cluster II)	Mean	135	1.66	1.35	-	0.21	0.33	79.94
	SE		0.01	0.00	-	0.00	0.00	
*M. tetraphylla* (Cluster III)	Mean	133	1.47	1.26	-	0.16	0.24	70.86
	SE		0.01	0.00	-	0.00	0.00	
Admixture	Mean	15	1.53	1.36	-	0.22	0.34	70.18
	SE		0.01	0.00	-	0.00	0.00	
Total	Mean	302	1.31	1.27	-	0.16	0.26	61.25
	SE		0.01	0.00	-	0.00	0.00	12.61

**Table 2:** Pairwise population matrix of Nei’s genetic distance (D) among the clusters of Macadamia wild germplasm, using SNP and silicoDArT markers.

Group	*M. integrifolia*	*M. tetraphylla*	*M. ternifolia/M. jansenii*
**SNPs**			
*M. tetraphylla*	0.20		
*M. ternifolia*/*M. jansenii*	0.27	0.23	
Admixture	0.06	0.07	0.22
**silicoDArTs**			
*M. tetraphylla*	0.17		
*M. ternifolia/M. jansenii*	0.17	0.16	
Admixture	0.08	0.05	0.16

**Table 3:** Summary statistic of AMOVA analysis in Macadamia germplasm using SNP and silicoDArT markers. df = degrees of freedom, SS = sum of squared observations, MS = mean of squared observations, Est. Var = estimated variance, % Var. = percentage of total variance. PhiPT = var. among groups (species)/ (var. among groups + var. within group).

Source	df	SS	MS	Est. Var.	% Var.	PhiPT Statistic	*p*-Value
**SNPs**							
Among species	3	175,814	58,604	951	45%		
Within species	298	344,429	1155	1155	55%		
Total	301	520,243		2107	100%	0.45	0.001
**silicoDArTs**							
Among species	3	74,996	24,999	401	34%		
Within species	298	235,037	789	789	66%		
Total	301	310,033		1190	100%	0.34	0.001

## Data Availability

The SNP and silicoDArT markers generated and analysed during this study are obtainable from The University of Queensland’s Institutional Data Access/Ethics Committee, but restrictions apply to the availability of these data. The dataset “DArTseq markers of wild macadamia species” is available at https://doi.org/10.14264/uql.2018.395, for researchers who meet the criteria for access to confidential data. Contact data@library.uq.edu.au.

## Supplementary Materials

The following are available online at https://www.mdpi.com/2223-7747/9/6/714/s1, Table S1. Summary of markers; Table S2. List of SNP markers; Table S3. List of silicoDArT markers; Table S4. Q value at K = 3, Table S5. Genetic distance of *M. integrifolia*; Table S6. Genetic distance of *M. tetraphylla*; Table S7. 302 accessions information.
